# Taxon-specific responses to different forestry treatments in a temperate forest

**DOI:** 10.1038/s41598-018-35159-z

**Published:** 2018-11-19

**Authors:** Zoltán Elek, Bence Kovács, Réka Aszalós, Gergely Boros, Ferenc Samu, Flóra Tinya, Péter Ódor

**Affiliations:** 1 MTA Centre for Ecological Research, Institute of Ecology and Botany, Vácrátót, Hungary; 2 MTA Centre for Agricultural Research, Agricultural Institute, Budapest, Hungary; 3 MTA-ELTE-MTM Ecology Research Group, Budapest, Hungary; 4 MTA Centre for Ecological Research, GINOP Sustainable Ecosystem Research Group, Tihany, Hungary; 5 Department of Plant Systematics, Ecology and Theoretical Biology, Institute of Biology, Eötvös Loránd University, Budapest, Hungary; 6 Szent István University, Department of Zoology and Animal Ecology, Gödöllő, Hungary

## Abstract

There are only few studies that explore the ecological consequences of forest management on several organism groups. We studied the short-term effects of four forestry treatments including preparation cutting, clear-cutting, retention tree group and gap-cutting in a temperate managed forest on the assemblage structure of understory plants, enchytraeid worms, spiders and ground beetles. Here we show, that the effect of treatments on the different facets of assemblage structure was taxon-specific. Clear-cutting and retention tree group strongly impoverished enchytraeids assemblages. Even if the species richness and cover of plants increased in clear-cutting and gap-cutting, their species composition moderately changed after treatments. For spiders only their species composition was influenced by the treatments, while the response of ground beetles was slightly affected. Short-term effect of forest management interventions on biodiversity might be compensated by the dispersal (spiders, ground beetles) and resilience (plants) of organism groups, however sedentary soil organism showed high sensitivity.

## Introduction

The majority of European forests are strongly modified due to anthropogenic disturbances, and only 4% of these can be considered as undisturbed, i.e. still dominated by natural processes and structures^1^. In these forests rare coarse-scale disturbances such as windthrows or forest fires and frequent fine-scale disturbances such as spontaneous gap dynamics may support the structural heterogeneity either at the stand- or at the landscape level^2^. The progressive technical development in forestry and the recurrent disturbance caused by rotation forest management had increasing negative effects on biodiversity^2–4^. European forests managed by rotation forestry systems are characterized by even aged stands, low tree species richness, homogenous structure and absence of a variety of tree-related microhabitats such as large dead logs or veteran trees. However, many of these managed forests may preserve high biodiversity including thousands of forest specialist species, large carnivores and special habitat types^5^. In the last decades, new initiatives fostered silvicultural approaches that, besides supporting timber production, may also aim to sustain natural processes and biodiversity. Such aims are reached, for instance, by the maintenance of old-growth attributes^6,7^, continuous cover forestry, retention forestry^8,9^ and forest management mimicking natural disturbances^2^. It is therefore crucial to explore how timber production can be harmonized with biodiversity conservation in forest management.

Although there are many case studies concerning the specific effects of various forest management treatments, there are only few initiatives in the temperate zone^10^ that compare experimentally the effect of multiple forestry treatments on biodiversity applying a multi-taxa approach. Previous meta-analyses explored the effect of certain management types on the diversity of specific organism groups^11^ or compared managed versus unmanaged stands^12^.

Most evidence points to the variability of responses within and across taxa^13,14^, suggesting that organism groups strongly related to specific microhabitats or structural conditions are influenced negatively by forest management^12,15,16^.

Vascular plants, for instance have equivocal responses to increasing forest management intensity^17^. In many cases their alpha diversity and cover showed positive response to management and was higher in intensively managed forests than in old-growth ones, because management usually increased light availability, which is a limiting factor for many species^12,18,19^. However, following the higher structural complexity, beta and gamma diversities were generally higher in old-growth than in managed stands^19,20^. Management intensity also effects species composition favouring non-forest, light demanding species, whereas many shade tolerant ancient woodland species disappeared after clear-cutting^21–23^.

Animals are more mobile and less resilient than plants, thus they can respond very fast to the alterations of habitat conditions. The effect of forest management on true soil organisms has been rarely studied, even though these organisms, such as enchytraeid worms (Annelida: Clitellata: Enchytraeidae) often play a crucial role in litter decomposition^24^. Changes in soil conditions such as moisture and temperature may mediate management effects on these groups of organisms^25^. A previous study revealed slight management effects on the abundance and composition of these worms in boreal forests, suggesting a beneficial effect of gap-cutting on their abundance as compared to closed stands^26^. Ground-dwelling arthropods such as spiders (Aranae) and ground beetles (Coleoptera: Carabidae) are less bound to the substrate and are more mobile, occupying a large variety of niches^27^. They respond to stand structural complexity at various temporal and spatial scales^28^ and they are strongly influenced by natural and anthropogenic disturbances^29–31^. They have been suggested as bioindicators of commercial forestry management^32,33^, being sensitive to environmental factors such as temperature, humidity and vegetation structure^34–37^.

To compare the effect of different forest management approaches on biodiversity, we studied the response of a range of organism groups with varying sensitivity to site conditions modified by management. In a randomised block experiment we investigated the short-term effect of four forestry treatments (each with a control - C) commonly used in rotation forestry system (preparation cutting - P, clear-cutting - CC, retention tree group - R) and selection forestry system (gap-cutting - G) on four organism groups before- and two years after implementation. Our major aim was to explore how the assemblages of these organism groups (composition, species richness and abundance) react to the different treatments. We also recorded the microclimatic conditions in the treatments, which enable to link the responses of the assemblages to the treatment induced by environmental changes.

Our hypotheses were the followings: (i) for all studied community parameters (species composition, species richness and abundance) we may expect strong responses from enchytraeid worms due to their increased sensitivity for environmental parameters by their relatively low mobility. For plants, the short term response will be intermediate due to the limited growth rate of newly established plants and survival of the original vegetation. Spiders and carabids might be less sensitive to treatment effects due to their higher mobility as compare to other studied organisms. In relation to treatment types, (ii) we expect the strongest responses of all assemblages in clear-cutting because it causes the most drastic changes of forest site conditions. Retention tree group (in the clear-cuts) can buffer more effectively light condition than temperature changes, thus we are expecting stronger responses from animals (mainly soil organisms) than from plants. Gap and preparation cutting show intermediate conditions between control and clear-cutting so we are expecting intermediate responses here for all studied organisms. However, the more open condition in gap cutting may lead to more extensive changes for plants than for animals.

## Results

### Assemblage composition

**Figure 1:**
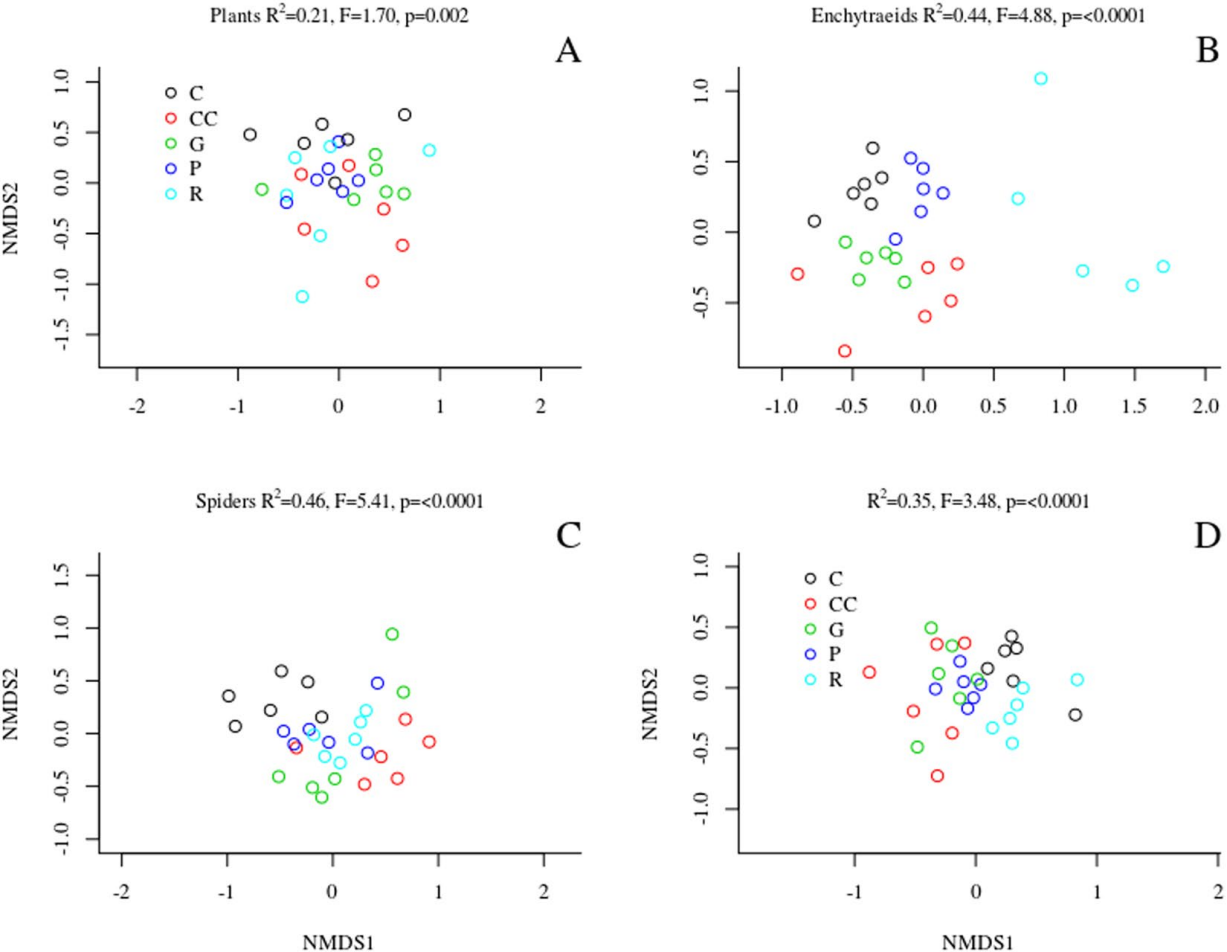
Ordination of the non-metric multidimensional scaling based on the abundance (cover for plants) of the studied organism groups. The compositional difference between treatments expressed as the results of the PERMANOVA (coefficient of determination, F and p values) are portrayed on each graph panel.

**Figure 2:**
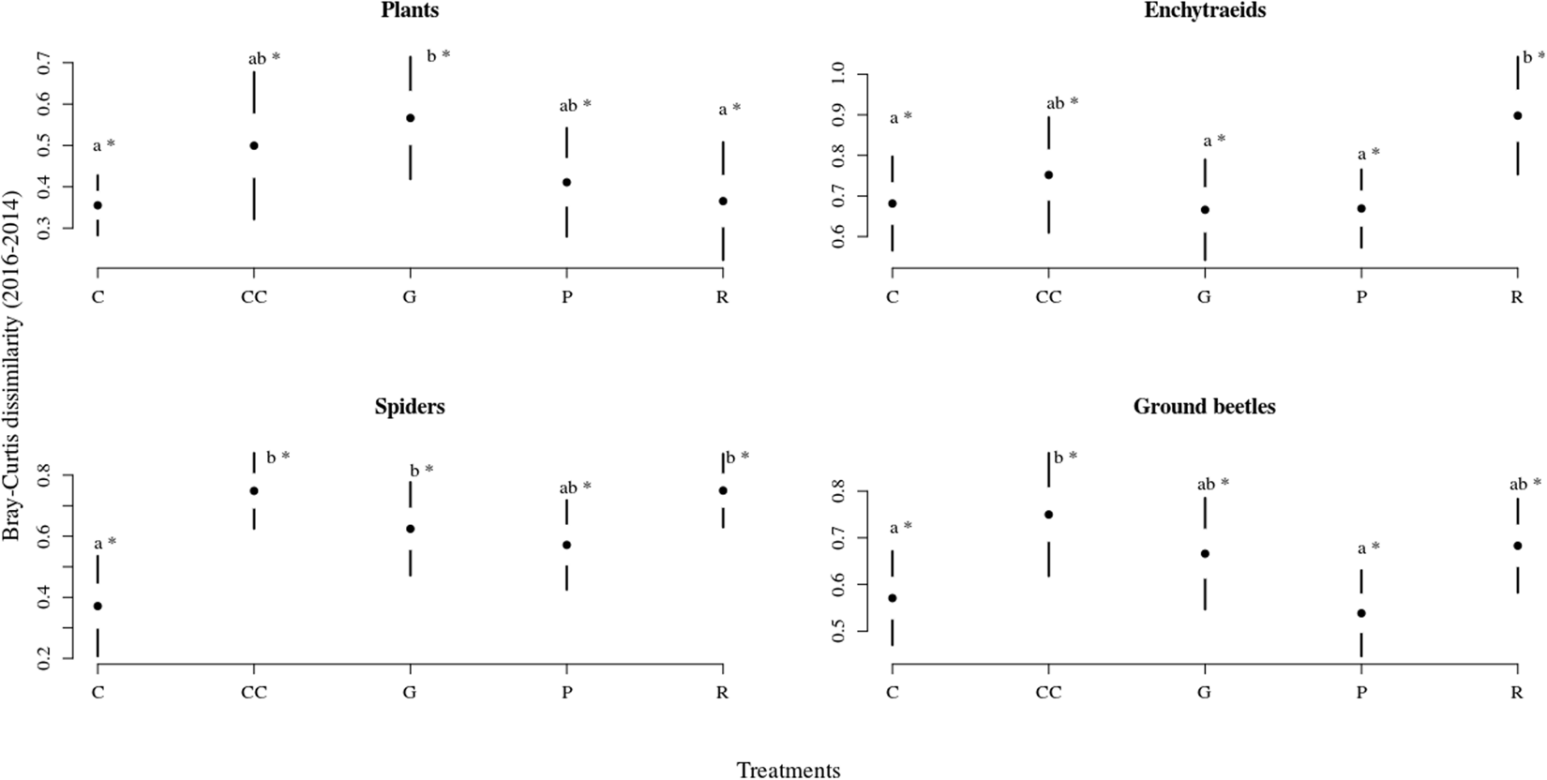
Casual changes in Bray-Curtis dissimilarity of the studied organism groups among treatments between 2014–2016. Full circles shows the mean, white space between the circles the standard error for mean, while vertical lines denote the standard deviations. The stars denote the real difference from zero (value of 2014) based on a regression through the origin, the letters designate the significant differences among treatments, significance level were set at 0.05 for both cases.

We observed a strong compositional difference in the case of enchytraeids and spiders, and a slight, but still significant difference in the case of plants and ground beetles across treatments (Fig. 1, Table 1). For plants (Fig. 1A), the plots representing different treatments overlapped considerably in the multivariate ordination space. When comparing the difference in species composition of plants across treatments, measured as the Bray-Curtis (BC) dissimilarity of species composition of a plot before and after treatments, we found that the gap-cutting was significantly different from control and retention tree group (Fig. 2). The enchytraeid worms of retention tree group separated from other treatments, while gap-cutting and clear-cutting (bottom of Fig. 1B) diverged from control and preparation cutting (upper part of Fig. 1B). Similar pattern was revealed by BC dissimilarities, the retention tree group was significantly different from gap-cutting, preparation cutting and control (Fig. 2). Spider assemblages in treatment plots were separated in the ordination space from those in control plots (Fig. 1C). Assemblages in the clear-cutting plots were the most distinct from control. Spider species composition in other treatments was intermediate between control and clear-cutting, largely overlapping with each other. The dissimilarity analysis showed that the control was different from all the other treatments, expect preparation cutting (Fig. 2). For ground beetles the plots of control, retention tree group and preparation cutting formed relatively homogeneous groups, but the treatments showed considerable overlap (Fig. 1D). The BC dissimilarities of control and preparation cutting were significantly lower than those of other treatments (Fig. 2). The compositional changes were significantly different between 2014 and 2016 in each taxa (Fig. 2).

**Table 1:** Bray-Curtis dissimilarity responses of the selected organism groups for different forest treatments based on GLMM.

*Groups*	*Variables*	*F*	*d.f*.	*p*
Plants	intercept	145.820	1,20	<0.0001
	treatment	3.531	4,20	0.0246
Enchytraeids	intercept	673.803	1,20	<0.0001
	treatment	4.236	4,20	0.0120
Spiders	intercept	558.083	1,20	<0.0001
	treatment	7.218	4,20	0.0009
Ground beetles	intercept	1028.548	1,20	<0.0001
	treatment	3.7067	4,20	0.0205

### Species richness

**Figure 3:**
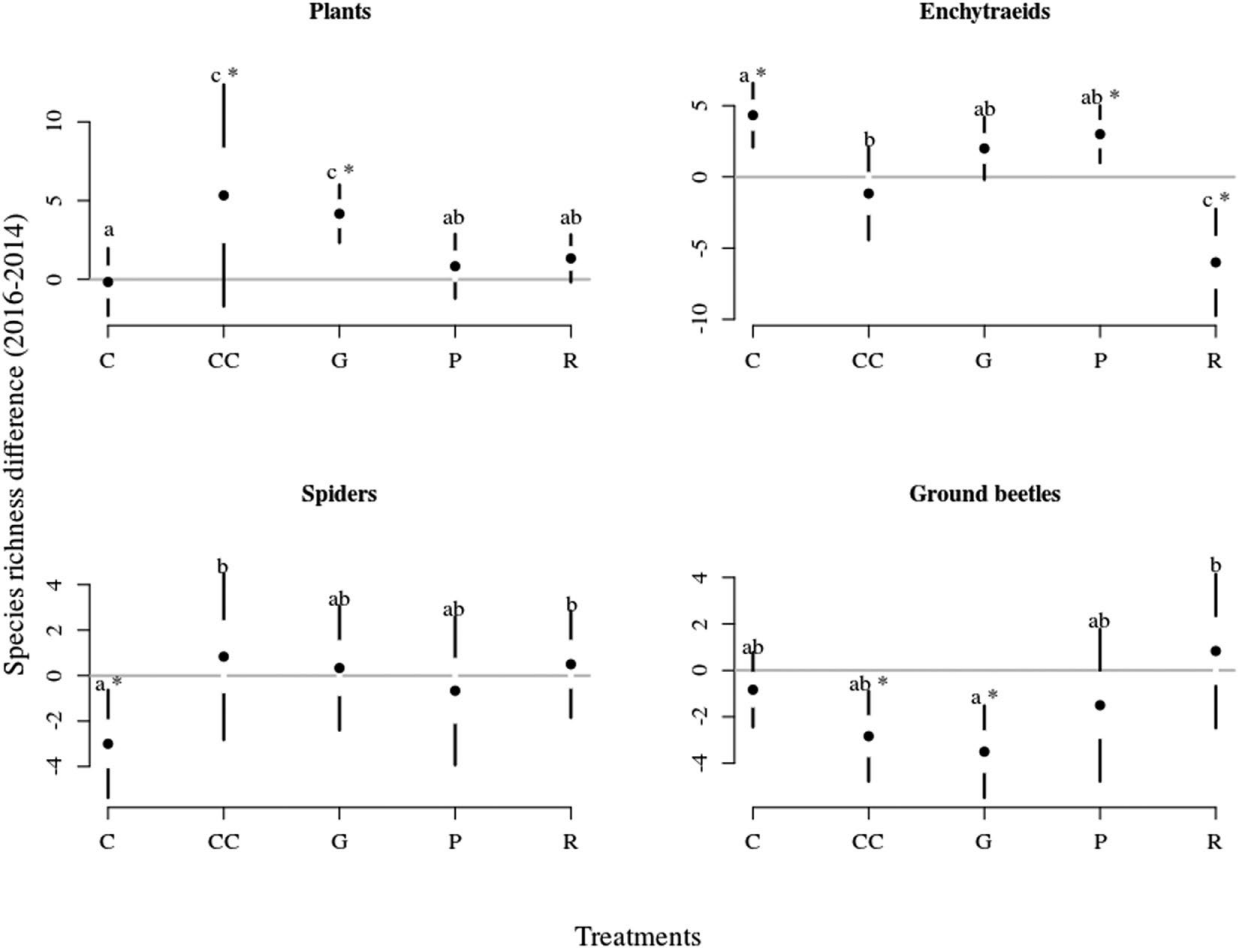
Equivocal changes in species richness of the organism groups among treatments between 2014–2016. Full circles shows the mean, white space between the circles the standard error for mean, while vertical lines denote the standard deviations. The stars denote the real difference from zero (value of 2014) based on a regression through the origin, the letters designate the significant differences among treatments, significance level were set at 0.05 for both cases.

The effect of the treatments for species richness was the strongest for enchytraeids, intermediate for plants and slightly significant for spiders and ground beetles (Table 2). Plants’ species richness increased in clear-cutting and gap-cutting two years after the treatments, whereas it did not change in control, preparation-cutting and retention tree group (Fig. 3). Enchytraeids’ species richness increased in control and preparation-cutting during the experiment (significant year effect), while it was the lowest in retention tree group. The species richness of spiders was not different between years or treatments, except for the control treatment, where a significant drop in species richness could be observed from 2014 to 2016. The number of ground beetle species decreased in clear-cutting and gap-cutting between years, while it was the significantly the highest in the retention tree group.

**Table 2:** Effect of treatments on the species richness difference between 2014 and 2016 for the organism groups based on GLMM.

*Groups*	*Variables*	*Chi-square*	*d.f*.	*p*
Plants	intercept	17.666	1	<0.0001
	treatment	23.365	4	0.0001
Enchytraeids	intercept	536.759	1	<0.0001
	treatment	33.566	4	<0.0001
Spiders	intercept	16.216	1	<0.0001
	treatment	10.035	4	0.0398
Ground beetles	intercept	122.445	1	<0.0001
	treatment	12.459	4	0.0142

### Abundance

**Figure 4:**
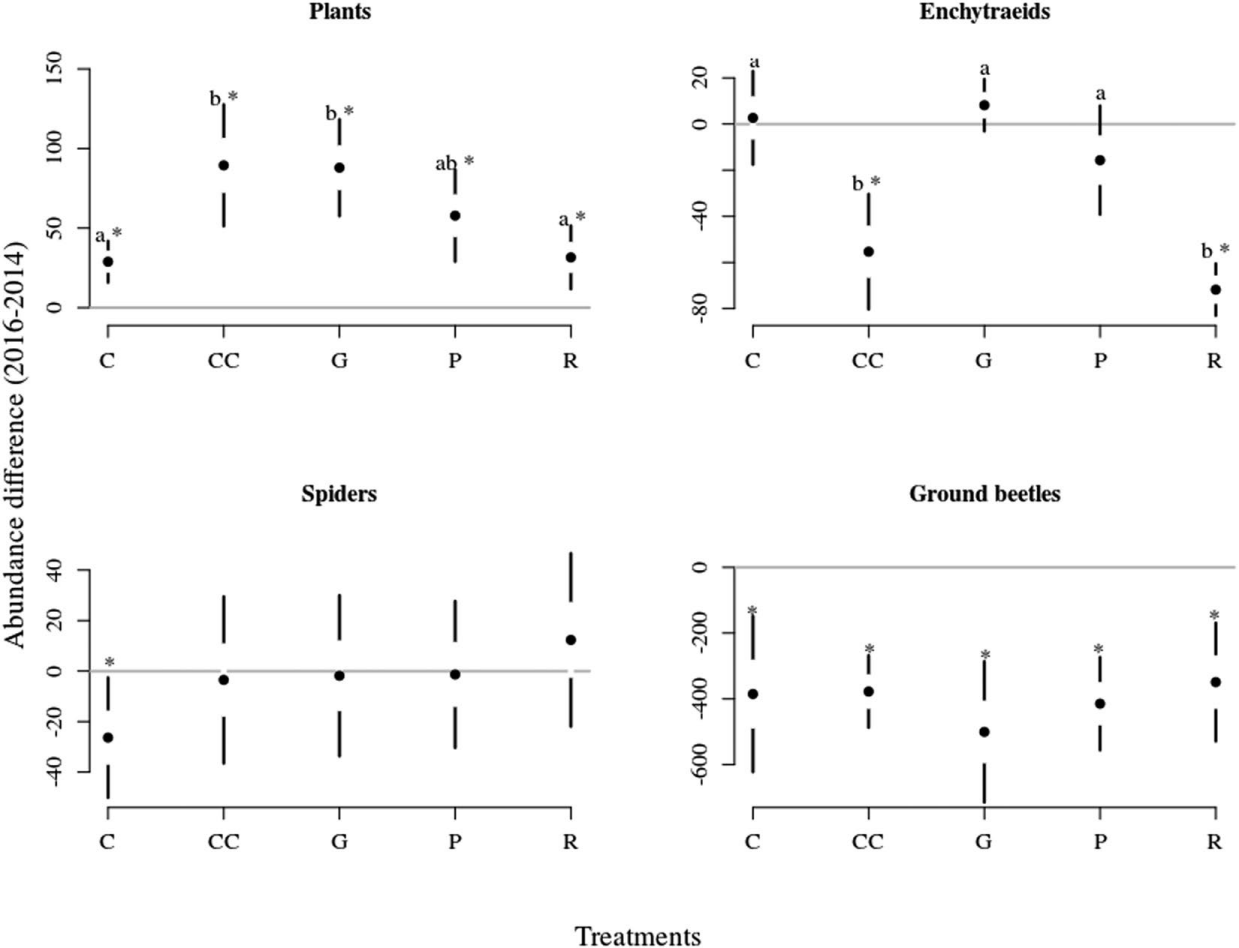
Contrasted changes in abundance (cover for plants) of the organism groups among treatments between 2014–2016. Full circles shows the mean, white space between the circles the standard error for mean, while vertical lines denote the standard deviations. The stars denote the real difference from zero (value of 2014) based on a regression through the origin, the letters designate the significant differences among treatments, significance level were set at 0.05 for both cases.

The effect of treatments on between-years abundance differences was the strongest for enchytraeids, intermediate for plants and was not significant for spiders and ground beetles (Table 3). There is an overall increase in the cover of plants in all treatments between years (Fig. 4). However the abundance was significantly higher in clear-cutting, gap-cutting and preparation cutting than in other treatments. There was a concordance in the response of enchytraeids between the years and treatments, their abundance significantly decreased in clear-cutting and retention tree groups for both effects. The only change in the abundance of spiders was a decline between years in the control treatment. Although there is an overall decline in the abundance of ground beetles between years, the treatment effect was not significant.

**Table 3:** Effect of treatments on the abundance (cover for plants) difference between 2014 and 2016 for the organism groups based on GLMM.

*Groups*	*Variables*	*F*	*d.f*.	*p*
Plants	intercept	56.3325	1	<0.0001
	treatment	10.7135	4	0.0001
Enchytraeids	intercept	47.2613	1	<0.0001
	treatment	18.6303	4	<0.0001
Spiders	intercept	0.5447	1	0.4680
	treatment	1.257	4	0.3238
Ground beetles	intercept	91.8165	1	<0.0001
	treatment	0.7181	4	0.5890

## Discussion

The integration of biodiversity conservation in forest management (and its planning) may requires effective monitoring of spatial and temporal changes of forest biotas including selected organisms with management-specific responses. Although this paper focused on the direct effects of treatments on the communities, these treatments affects through the changes of environmental conditions, which are detailed in electronic supplement (ESM-Fig. 2). The strongest environmental changes were detected in clear-cuttings: light conditions considerably increased, air temperate increased, air humidity decreased with high diurnal variance, soil temperature and humidity also increased. Gap-cutting is characterised by high light conditions, but buffered air temperature and humidity, it has the highest soil moisture and lowest soil temperature among treatments. In retention tree group light conditions were similar as in the control, but air temperature and humidity was similar to the clear-cutting (only the diurnal variance was buffered), it is characterised by low soil moisture and high soil temperature. Preparation cutting was the most similar to the control, it showed only slight increase in light, air and soil temperature. Considering all groups, the strongest treatment effects were detected in clear-cuttings which can be expained by the drastic changes in all environmental variables (ESM-Fig. 2). Gap-cutting had a positive effect on the herbaceous vegetation, possibly as a result of the increased ground-floor light availability. Retention tree groups where soil moisture decreased and soil temperature increased considerably, had a negative effect on sedentary enchytraeids, but a slightly positive effect on ground beetles. Preparation-cutting, which caused only moderate environmental changes, brought limited changes in the studied organism groups. In addition, these effect of treatments on the studied organisms group might be explained by their taxon-specific mobility. This was best exemplified by the sedentary, non-resilient enchytraeids, for which all the three studied community parameters changed considerably. The cover of non-mobile plants was affected, but their species composition responded weakly. In ground beetles, which have moderate mobility and resiliency, richness and composition showed relatively weak responses. In spiders, which can be regarded the most mobile group, species composition changed gradually along the management intensity gradient from clear cutting toward control treatments, while species richness and abundance showed difference between control and all other treatments.

### Organism specific assemblage responses to management

Sustainable forest management demands a better understanding of the environmental drivers affecting assemblage composition and diversity across taxonomic groups to ensure the multifunctionality of forests^38,39^. Thus, it is important to explore the interactions between forestry treatments and the different taxonomic groups unravel possible ecological consequences. We found that plants and enchytraeids provided the most drastic response two years after the implementation of the treatments. Thus, we suggest that these groups can act as early warning signalers of undesired effects of forest management due to their short-time response. The direction of changes of the two groups was different: in clear-cutting the richness and abundance of plants increased while those of enchytraeids decreased, retention tree group did not seem to affect plants but had a strong negative effect on enchytraeids. It can be assumed that the apparently low dispersal capabilities of enchytraeids make this group especially sensitive to habitat alteration, because local extinctions are not compensated by immigration. Previous findings^26^, for instance, proved that enchytraeid density responded negatively to forestry treatments in Finland, however, three years after the implementation some management-specific density increase was observed. This study also pointed out that decomposer communities such as enchytraeids are not solely controlled by soil resources, but also by abiotic soil conditions which are greatly affected by forest management^26^.

The species composition of plants, changed slightly, which is possibly linked to the presence of perennial species and to the seed bank in the soil. However, the improved light conditions and soil moisture in treated plots might lead to an increased cover of existing species, and to the appearance of immigrant and dormant species, resulting in an increase in cover and species richness^40^. The sensitivity of spiders and ground beetles to forest management was inconsistent; the abundance of both groups was mostly unaffected by the treatments; however, over the observation period a general decline in abundance was detected for both groups. Previous results^41^ demonstrated that the high mobility of orthopteran species can mask spatial heterogeneity between habitats, whereas sedentary species contributed more to maintaining beta diversity between habitats. We may argue that similarly to the orthopteran study, the high mobility of spiders and ground beetles might mask the response to forest management, a pattern that can be identified in the almost equal abundance in the various treatments.

### Management affects organism groups through different environmental variables

Plant understory often responds strongly to forest management^37,42,43^, because management practices change the microclimatic conditions of the forest habitat. These patterns may explain the high species richness and cover of plant understory in clear cutting and gap cutting, as well as the high cover in preparation cutting. We found that clear-cutting and gap-cutting had the highest light and soil moisture levels (ESM-Fig. 2), while soil temperature and vapor pressure deficit was the highest in the clear-cutting as compared to other treatments. This is in agreement with a previous study^42^ which suggests that post-treatment microclimatic conditions might be responsible for changes in plant communities, chiefly for the appearance of non-forest plants in clear-cuts. We argue that in spite of these changes the weak differences in the species composition of plants in relation to management can be explained by the survival of perennial species^44^. Similarly to plant communities, we found that the enchytraeids responded promptly to the treatments and associated changes in environmental parameters. Drastic increase in soil temperature (ESM-Fig. 2) in retention tree group and clear-cutting treatments may contribute to the rapid decrease in both spcies richness and abundance in this strictly sedentary group^26^. However these issues may require further investigations as two recent global meta-analyses revealed that there is no evidence that retention forestry in temperate forests would be a safeguard of biodiversity^9,45^, however one of the major function of this treatment type to conserve the native forest biotas for the recolonisation of the logged area during forest regeneration. In addition, these meta-analyses also suggest that the size and the spatial arrangement of retention trees may influence the effectiveness of this treatment, in which regard further investigations might be necessary.

Ground-dwelling predatory arthropods are among the best indicators of forest management^37,43^. These groups have a short lifespan, have a higher position in the food web and give a complex response to changes in their abiotic and biotic environment^35^. These groups give a good example that evoking environmental filtering is often insufficient to explain assemblage responses. As argued in a recent opinion paper^46^, abiotic environment determines assemblage composition not only directly via survival, but also by affecting biotic interactions. Spider abundance and species richness did not change significantly in the treatments, however their species composition was very sensitive to treatments induced environmental changes. Previous studies showed that spider assemblages react quickly to changes in vegetation structure^28,47,48^ and vascular plant richness being species turnover enabled by the high mobility of spider species. Ground beetles, a group with moderate mobility, gave weaker and somewhat less specific response manifesting in a general decline in abundance. This is presumably caused by the short-term response of these invertebrates, which is explanied by the loss of forest specialists due to treatments^36^. Ground beetles reacted positively to the retention group treatment, where the higher species richness of ground beetles might be explained by the appearance of open-habitat species^36^. Some recent findings^38^ revealed that functional diversity of ground beetles are not influenced only by the diversity of forest ground vegetation, other indirect drivers may occur. All issues mentioned above prompted us to emphasize that treatment effect was the strongest in retention tree groups and clear-cutting for all studied organisms in the present study.

### Conclusions

Multiple ecosystem functions in forests require sets of species forming communities. Particularly in the light of global climatic change scenarios, which predict more frequent disturbances and extreme climatic events, it is important to explore the relationship between biodiversity and forest management practices in forestry since the forest can be a tool for mitigation of climate change^49^. Yet, the majority of research on the impact of forestry on biodiversity has focused on a specific relation between a certain management type and the response of a selected organism group. Although our results do not provide direct evidence, we suggest that dispersal dynamics may play a crucial role in the taxon-specific responseto various forest management practices. Enchytraeid worms, the least mobile animal group in the study, gave the most pronounced short-term response to the treatments. Thus, from all studied invertebrate organisms, they can be candidate early-warning signalers in Central European forests. In contrast, the more mobile ground-dwelling predatory arthropods, spiders and ground beetles, did not exhibit any easily interpretable mass effect, presumably due to their quick colonization by dispersal which enabled them to adapt to changes in the forest environment. In our multi-taxon approach we could identify organism groups that gave easily detectable mass response, while others gave taxon-specific responses, that might allow the detection of slight environmental changes. Such as in the present study, where we could prove that gap-cutting had the least adverse effect on forest dwelling organisms, the approach presented here might be useful in monitoring the ecological effects of various forestry treatment practices simultaneously. These results can contribute to the assessment of forest management’s effect on biodiversity in Central European oak-hornbeam forests. However there are sporadic information available about this forest type and its region, which demands further investigations in the future.

## Methods

### Study area

The study area of the Pilis Experiment was in the vicinity of Pilisszántó village in the Pilis Mountains (N47°40′ and E18°54′), Northern part of Hungary (Supplementary Fig. 1). The elevation is 370–470 m a.s.l., average annual mean temperature is 9.0–9.5 °C, mean annual precipitation of 600–650 mm^50^. The bedrock consists of limestone and red sandstone with loess, the most common soil type is lessivage brown forest soil (luvisol). The experiment was carried out in a structurally homogenous, 80 years old, 40 ha sized, managed sessile oak-hornbeam forest stand^5^ (Natura 2000 code: 91G0^5^). The stand was cut many times in the past, recently it has been managed by shelterwood silvicultural system resulting into an even-aged, structurally homogenous stand. The canopy layer is dominated by sessile oak (*Quercus petraea* (Matt.) Liebl.), and the mean tree height is 21 m, mean diameter (at breast height) is 28 cm, subordinate species of this layer are turkey oak (*Quercus cerris* L.), beech (*Fagus sylvatica* L.) and wild cherry (*Prunus avium* L.). Hornbeam (*Carpinus betulus* L.) forms a 11 m height secondary canopy layer with manna ash (*Fraxinus ornus* L.) appears as a subordinate species. The shrub layer is scarce; the understory cover is approximately 30%, consisting mainly of mesic forest plants, dominated by *Carex pilosa* Scop. and *Melica unflora* L.

### Experimental design

Four treatments plus control were established between December 2014 and January 2015 in the forest stand following a randomized complete block design with six-replicate blocks (Supplementary Material Fig. S1). The implemented treatments were the following: 1. Control (C): closed canopy stand without any treatment; 2. Clear-cutting (CC): a 0.5 ha circular clear-cutting area of 80 m diameter, surrounded by closed canopy stand; 3. Gap-cutting (G): an artificial circular gap in the closed stand (20 m diameter, approx. one height/diameter ratio); 4. Preparation cutting (P): 30% of the total basal area of the dominant tree layer and the whole secondary tree layer was removed in a spatially uniform way in a circle of 80 m diameter; 5. Retention tree group (R): a circular group of upper canopy trees (20 m diameter, 8–12 dominant individuals, untouched subcanopy layer) was retained in the clear-cutting. Clear-cutting, retention tree group and preparation cutting represent characteristic stages of rotation forestry system, while gap cutting is often implementation of continuous cover forestry (selection forestry system). Altogether 30 plots were studied representing five treatments and replicated in six blocks (Supplementary Material Fig. S1). We considered as a combinations of treatments and blocks as a basic sampling units.

### Data collection

Data collection followed the concept of Before After Control Impact experiments^51^, recording all investigated variables in the vegetation period of 2014 (before the implementation) and in 2016 (two years after the implementation).

The microclimatic conditions (photosynthetic active radiation, relative diffuse light, air temperature, air humidity, vapor pressure deficit, soil temperature, soil water content) of the treatments were systematically measured before and after the implementation of the treatments. The methodology and major results are detailed in the Electronic Supplementary Material (Fig. S2).

The surveys of vascular plants was carried out in a 2 × 2 m sized quadrat in each plot within a fenced 6 × 6 m area excluding the effect of grazing. The cover of species was estimated in percentage, only arboreal individuals under 0.5 m height were included in the sampling. The survey was carried out in spring (April) and summer (June). Data of the two aspects were merged using the maximum cover values of each recorded species.

We sampled enchytraeids (Annelida: Enchytraeidae) through soil samples taken with a soil corer (diameter 5 cm, depth: 12 cm resulting 235 cm^3^ sample volume). Three samples were taken in the plots in spring and autumn, every year, and mixed into an average sample (ca. 235 cm^3^), the worms were extracted by wet funnel method^52^. The datasets from the two sampling occasions were pooled by year.

To sample ground dwelling predatory arthropods (spiders (Aranae) and ground beetles (Coleoptera: Carabidae)), four pitfall traps were installed in every plot around the fenced area in each direction. Two sampling intervals (one month in spring and one in autumn) was set, corresponding to the highest activity regime of the beetles^53^ and spiders^54^. The traps were made of 85 mm diameter plastic cups; each containing approximately 250 cm^3^ of a 50% solution of propylene glycol and water, saturated with salt and with a drop of odorless detergent to reduce surface tension. A dark green plastic roof protected the solution from litter and rain. The data of the pitfall traps of the same plots were merged resulting 30 sampling units for both study years. The traps were checked monthly.

### Ethical Approval

The observation of plant communities in this study were non-invasive, while the field sampling of invertebrates such as enchytraeid worms, spiders and ground beetles were conducted under the license from the respective Hungarian authority (Közép-Duna-Völgyi Környezetvédelmi és Természetvédelmi Felügyelőség KTF:30362-3/2014).

### Data analysis

Non-metric Multidimensional Scaling (NMDS) and Permutational Multivariate Analysis of Variance (PERMANOVA) with square-root transformation was applied to test for dissimilarities in species composition among treatments using the Bray-Curtis index of dissimilarity^55^. Data before and after treatments were analyzed separately, but only the post-treatment analyses (dataset from 2016) are presented here. In order to explore the direction of changes in assemblage composition, we calculated Bray-Curtis dissimilarities between the same plots in 2014 and 2016, the effect of treatments on the dissimilarities were compared using Generalized linear mixed-effect models^56^ (GLMM) as described below.

The (signed) differences between 2014 and 2016 for species richness and abundance (cover for plants) were used for all organism groups as response variables during the analyses. Positive values indicated increase, while negative values denoted a decrease in the variables after treatment implementation. GLMMs were used to explore the effect of treatments (considered as a fixed effect term) on species richness and abundance difference (as response variables) of the studied organisms, while blocks were used as a random factor. Two families of distribution were applied: “Poisson” for species richness data and “Gaussian” for abundance data and Bray-Curtis dissimilarities. The models were tested with the default Laplace approximation to the log-likelihood. The model diagnostics includes the inspection of model residual’s structure (Pearsons’s type) versus fitted values and degrees of freedom either in model’s output or in graphs (ESM Tables 1–3). For overdispersions, we also compared the Poisson vs. Quasipoisson fit for the same model structure; Gaussian distribution was revelaed by Shapiro-Wilk test and quantile-quantile plots. In case of significant treatment effects, the differences between treatment levels were evaluated by multiple comparisons (with Tukey computed contrast matrices for several multiple comparison procedures). We also tested the significance of changes (cited as year effect in results) between 2014 and 2016 (true difference in estimates from zero) for each treatment by repeating the models with exclusion the effect of intercept^57^.

The analyses were carried out in R 3.4.1^58^; using the package vegan^59,60^ for NMDS (function “metaMDS”) and PERMANOVA (function “adonis”), lme4^61^ for GLMM on species richness (function “lme”), nlme^62^ for GLMM on abundace and Bray-Curtis dissimilarities (function “nlme”) and package multcomp^63^ for multiple comparisons (function “glht”).

## Electronic supplementary material

Supplementary material

Dataset 1

Dataset 2

Dataset 3

Dataset 4

## Data Availability

All data generated or analyzed during this study are included in this published article (and its Supplementary Information Files).
