# Supplementary material for: Taxon-specific responses to different forestry treatments in a temperate forest
Source: Sci Rep. 2018 Nov 19;8:16990. doi: 10.1038/s41598-018-35159-z (PMC6243015; doi:10.1038/s41598-018-35159-z)
Supplement: Supplementary file 1 — Supplementary material [file 41598_2018_35159_MOESM1_ESM.pdf]

SUPPLEMENTARY MATERIAL - ZOLTÁN ELEK, BENCE KOVÁCS, RÉKA ASZALÓS, GERGELY BOROS, FERENC SAMU, FLÓRA TINYA & PÉTER ÓDOR: TAXON-SPECIFIC RESPONSES TO DIFFERENT FORESTRY TREATMENTS IN A TEMPERATE FOREST

ESM-Fig. 1: Map of the study area in the Pilis Mountains, Hungary between 2014-2016.

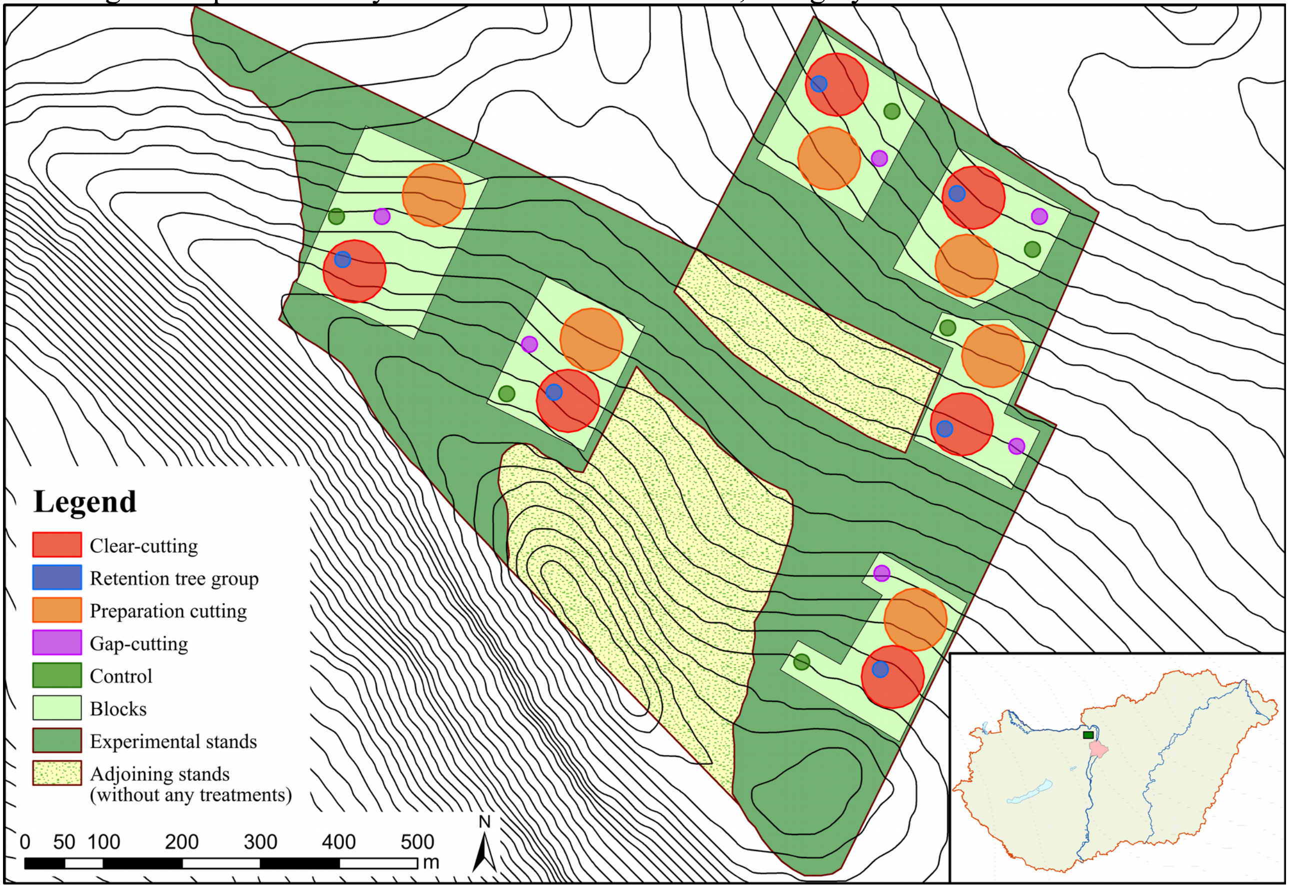

ESM-table 1: Summary of general linear mixed-effect models relating the forest treatments to differences in Bray-Curtis dissimilarities between 2014-2016 in Pilis mountains, Hungary. Legend: C-control, CC-clear-cutting, G- gap cutting, P-preparation cutting, R-retention tree group; significances are marked in bold.

Groups	*variables*	*estimated*	*SE*	*t*	*p*
Plants	**C (intercept)**	**0.355**	**0.056**	**6.285**	**<0.00001**
	**CC**	**0.144**	**0.068**	**2.107**	**0.04**
	**G**	**0.210**	**0.068**	**3.084**	**0.005**
	P	0.055	0.068	0.813	0.425
	R	0.01	0.068	0.148	0.883
Enchytraeids	**C (intercept)**	**0.681**	**0.07**	**9.631**	**<0.00001**
	CC	0.07	0.091	0.771	0.449
	G	-0.112	0.091	-1.234	0.231
	P	-0.026	0.091	-0.291	0.774
	**R**	**0.225**	**0.091**	**2.473**	**0.022**
Spiders	**C (intercept)**	**0.371**	**0.058**	**6.4**	**<0.00001**
	**CC**	**0.377**	**0.082**	**4.59**	**0.0001**
	**G**	**0.252**	**0.082**	**3.082**	**0.004**
	**P**	**0.200**	**0.082**	**2.439**	**0.02**
	**R**	**0.378**	**0.082**	**4.608**	**0.001**
Ground beetles	**C (intercept)**	**0.57**	**0.044**	**12.76**	**<0.00001**
	**CC**	**0.178**	**0.063**	**2.82**	**0.009**
	G	0.095	0.063	1.505	0.144
	P	-0.032	0.063	-0.512	0.613
	R	0.112	0.063	1.773	0.088

ESM-table 2: Summary of general linear mixed-effect models relating to the different forestry treatments to species richness difference between 2014-2016 in Pilis Mountains, Hungary. Legend: C – control, CC – clear-cutting, G – gap-cutting, P – preparation cutting, R – retention tree group; significant effects are marked in bold.

Groups	*variables*	*estimated*	*SE*	*z*	*p*
Plants	**C (intercept)**	**1.57**	**0.18**	**8.48**	**<0.0001**
	**CC**	**0.75**	**0.22**	**3.37**	**0.0007**
	**G**	**0.64**	**0.22**	**2.78**	**0.005**
	P	0.18	0.25	0.74	0.45
	R	0.27	0.24	1.09	0.27
Enchytraeids	**C (intercept)**	**2.59**	**0.11**	**23.168**	**<0.0001**
	**CC**	**-0.53**	**0.18**	**-2.89**	**0.003**
	G	-0.19	0.16	-1.15	0.24
	P	-0.1	0.16	-0.64	0.51
	**R**	**-1.49**	**0.28**	**-5.3**	**<0.0001**
Spiders	**C (intercept)**	**1.05**	**0.26**	**4.02**	**<0.0001**
	**CC**	**0.82**	**0.28**	**2.92**	**0.003**
	**G**	**0.74**	**0.28**	**2.62**	**0.008**
	**P**	**0.57**	**0.29**	**1.96**	**0.04**
	**R**	**0.77**	**0.28**	**2.72**	**0.006**
Ground beetles	**C (intercept)**	**1.81**	**0.16**	**11.06**	**<0.0001**
	CC	-0.39	0.25	-1.51	0.13
	**G**	**-0.56**	**0.27**	**-2.07**	**0.03**
	P	-0.11	0.23	-0.47	0.63
	R	0.23	0.21	1.08	0.27

ESM-table 3: Results of general linear mixed-effect models relating to the different forest treatment to differences in abundance/cover (for plants) between 2014-2016 in Pilis Mountains, Hungary. Legend: C-control, CC-clear-cutting, G-gap-cutting, P-preparation cutting, R-retention tree group; significances are marked in bold.

Groups	*variables*	*estimated*	*SE*	*t*	*p*
Plants	**C (intercept)**	**28.87**	**11.22**	**2.57**	**0.01**
	**CC**	**60.53**	**12.63**	**4.78**	**0.0001**
	**G**	**59.05**	**12.63**	**4.67**	**0.0001**
	**P**	**28.95**	**12.63**	**2.29**	**0.03**
	R	2.7	12.63	0.21	0.83
Enchytraeids	C (intercept)	2.66	7.93	0.33	0.74
	**CC**	**-58**	**11.22**	**-5.16**	**0.0001**
	G	5.5	11.22	0.48	0.62
	P	-18.33	11.22	-1.63	0.11
	**R**	**-74.46**	**11.77**	**-6.32**	**<0.0001**
Spiders	**C (intercept)**	**-26.33**	**12.49**	**-2.1**	**0.04**
	CC	22.83	17.66	1.29	0.21
	G	24.5	17.66	1.38	0.18
	P	25	17.66	1.41	0.17
	**R**	**38.66**	**17.66**	**2.18**	**0.04**
Ground beetles	C (intercept)	-385.16	74.41	-5.17	0
	CC	7.5	96.8	0.07	0.93
	G	-115.16	96.8	-1.18	0.24
	P	-29.5	96.8	-0.3	0.76
	R	36.16	96.8	0.37	0.71

Brief description of microclimate measurements and the major results

Systematic microclimate measurements were taken since March 2014 in the center of each fenced plot. Temporally synchronized data collections were carried out using HOBO data loggers (Onset Computer Corporation, Bourne, USA) mounted on wooden poles. In every month through the growing season (March-October), 72-hour logging periods were applied with 10-min logging intervals. Photosynthetically active radiation (PAR, λ=400-700 nm; $\mu Em^{-2}s^{-1}$) was measured at 150 cm above ground level; air temperature (T_{air}; °C) and relative humidity (RH; %) data were collected at 130 cm above ground level housed by radiation shields. Soil temperature (T_{soil}; °C) was measured at 2 cm below ground level. Soil water content (SWC; m^3/m^3) was gauged with soil moisture sensors buried 20 cm below ground level to measure the average soil moisture of the topsoil (10-20 cm). Air temperature and relative humidity data were used to calculate vapor pressure deficit (VPD; kPa) values at every logged occasion following the recommendations of Allen et al. (1998): $VPD=(0.6108)\{exp[17.27 \cdot T/(237.3+T)]\} \cdot (1-RH/100)$. The rationale of using VPD as a background variable is that it can give a direct indication of the atmospheric moisture conditions independently of the actual temperature, therefore it is a good standalone indicator of the atmospheric factors influencing evaporation (Anderson, 1936). Relative diffuse light (DIFN; %) was measured by LAI-2000 Plant Canopy Analyzer (LI-COR Inc., Lincoln, USA), in the center of each plot, at 130 cm above ground level. Measurements were carried out once a year (in July) at dusk to avoid direct light. For this variable repeated measurements are not needed (Tinya et al., 2009). Reference measurements (for calculating above-canopy light) were performed in an adjacent open field nearby.

In the case of the monthly repeated microclimatic variables, datasets were split into 24-hour subsets and based on these data means were calculated for each plot. For the analyses, relative data (i.e. synchronous differences from the control) were used avoiding the effects of the actual weather conditions. In this paper, only means were used for data analyses.

Following the suggested steps of data exploration by Zuur et al. (2010), e.g. dealing with outliers or data transformation if it was necessary, linear mixed models with maximum likelihood estimations were used to explore the effect of treatments and time (months) on the measured microclimate variables as response variables (Faraway, 2006). Blocks were used as random factor. Models' goodness-of-fit values were measured applying likelihood-ratio test-based coefficient of determination (R^2_{LR}; Bartoń, 2016). The differences between control and treatments were tested by random effects models (Zuur et al., 2009). In case of significant treatment effects, the differences between treatment levels were evaluated by multiple comparisons (pairwise Tukey HSD tests with alpha=0.05 among means; Hothorn et al., 2008).

Data analysis was performed with R version 3.4.1. (R Core Team, 2017). Mixed models were conducted by R package 'nlme' (Pinheiro et al., 2017), multiple comparisons were appraised by 'multcomp' (Hothorn et al., 2008), determination coefficients of the mixed models were calculated by 'rsquaredLR' function of 'MuMIn' package (Bartoń, 2016).

The amount total and diffuse light were both the highest in clear-cutting, in gap-cutting, light-increment was intermediate, whereas it was the lowest and very similar in the preparation cutting and retention tree group (ESM-figure 2.A and B). Nevertheless, in all cases light values were significantly higher than it was recorded in the control. Air temperature was the highest in clear-cutting followed by, in decreasing order, retention tree group, gap-cutting and preparation cutting (ESM-figure 2.C). Relative humidity was higher in gap-cutting, and it was very low in clear-cutting and retention tree group (ESM-figure 2.D). These values resulted in high vapor pressure deficit in clear-cutting and retention tree group compared to gap-cutting and preparation cutting (ESM-figure 2.E). We found significant increase in soil moisture in the gap-cutting and in a less degree in clear-cutting, while the driest treatment was the retention tree group due to the high VPD and drainage of the residual trees (ESM-figure 2.G). As SWC was highest in gap-cutting, it caused the lowest soil temperature-increment among the experimental treatments (ESM-figure 2.F).

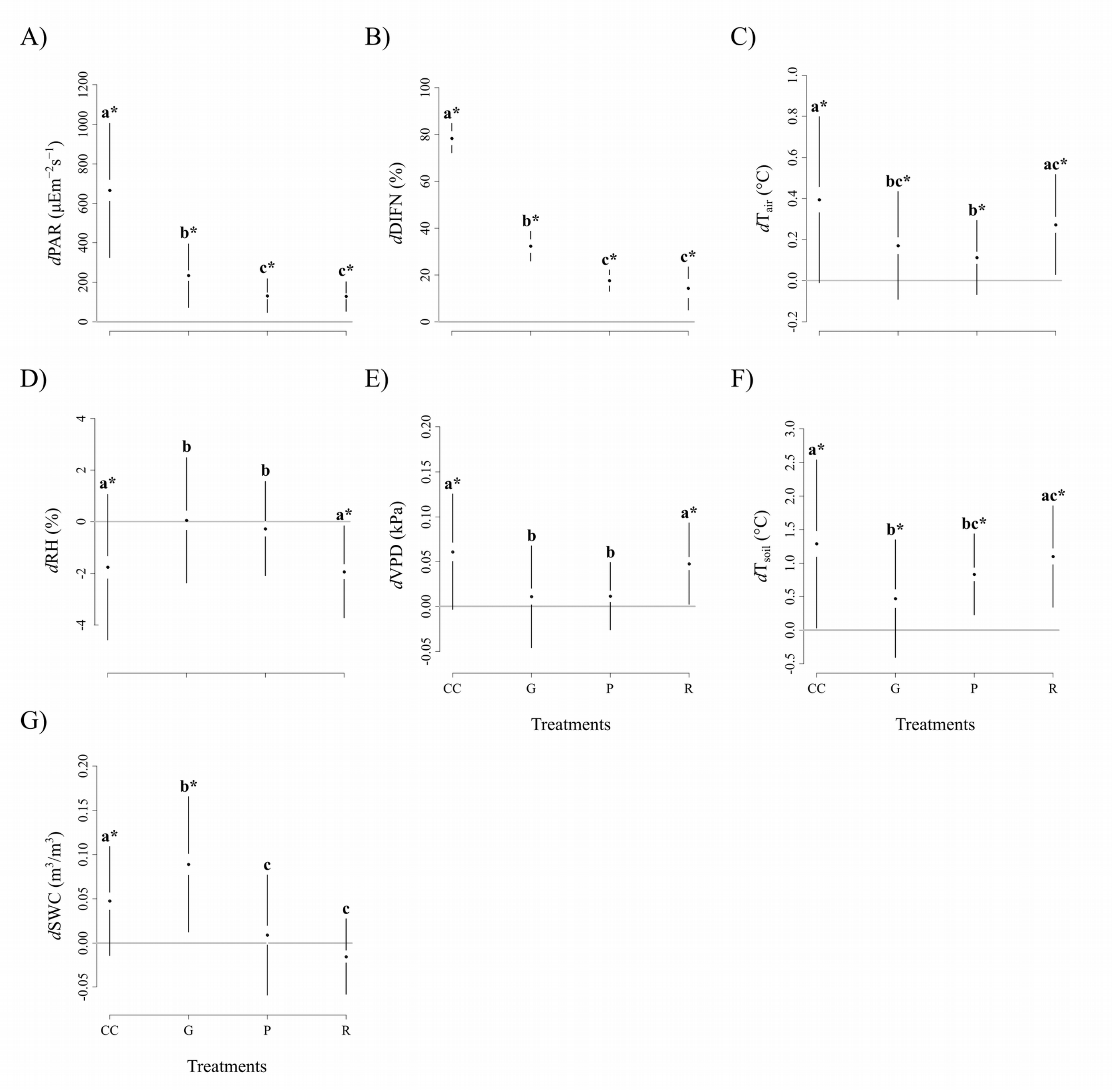

ESM-figure 2. Differences in microclimate variables among forestry treatments two years after the implementation of the experiment (2016) in Pilis Mountains, Hungary.

For all variables the values represent the mean differences from the treatment 'Control' for the whole growing season indicated by letter *'d'* in the variable abbreviations. A) PAR - photosynthetically active radiation; B) DIFN - relative diffuse light compared to open conditions; C) T_{air} - air temperature (°C); D) RH - relative humidity; E) VPD - vapor pressure deficit; F) T_{soil} - soil temperature; G) SWC - soil water content. Treatment codes: CC - clear-cutting, G - gap-cutting, P - preparation cutting and R - retention tree group. On the graphs, full circles show the mean, white belts between circles and vertical lines represent the standard error for the mean, while vertical lines denote the standard deviation of the samples. Letters designate the significant differences among treatments (pairwise comparisons based on the linear mixed models: Tukey-test, alpha=0.05), while asterisks denote significant differences from values measured at control plots (random intercept models, alpha=0.05).

According to the analyses, preparation cutting with the remained 70% of the original living stock is able to maintain microclimatic conditions of the closed forests the most. Albeit the high light increment and moderately increased temperature, in gap-cutting a humid microclimate and cool topsoil conditions are present. Clear-cutting shows the most drastic microclimatic changes, while retention tree group may buffer light conditions compared to clear-cutting, but it can be characterized by high soil and air temperature and low soil moisture conditions.

Literature cited:

Allen R. G., Pereira L. S., Raes D. & Smith M. (1998): Crop Evapotranspiration – Guidelines for Computing Crop Water Requirements. FAO Irrigation and Drainage Paper 56.

Anderson, D. B. (1936) Relative humidity or vapor pressure deficit. Ecology 17(2):277–282.

Bartoń, K. (2016). MuMIn: Multi-Model Inference. R package version 1.15.6. https://CRAN.R-project.org/package=MuMIn

Faraway, J.J. (2006): Extending the Linear Model with R: Generalized Linear, Mixed Effects and Nonparametric Regression Models (Texts in Statistical Science), Chapman and Hall/CRC.

Hothorn, T., Bretz, F. & Westfall, P. (2008): Simultaneous Inference in General Parametric Models. Biometrical Journal 50(3): 346-363.

Pinheiro, J, Bates, D, DebRoy, S, Sarkar, D & R Core Team (2017). nlme: Linear and Nonlinear Mixed Effects Models. R package version 3.1-131 https://CRAN.R-project.org/package=nlme

R Core Team (2017). R: A language and environment for statistical computing. R Foundation for Statistical Computing, Vienna, Austria. https://www.R-project.org/.

Tinya, F., Mihók, B., Márialigeti, S., Mag, Zs. & Ódor, P. (2009): A comparison of three indirect methods for estimating understory light at different spatial scales in temperate mixed forests. Community Ecology 10(1): 81-90.

Zuur, A.F., Ieno, E.N., Saveliev, A.A. & Smith, G.M. (2009): Mixed Effects Models and Extensions in Ecology with R (Statistics for Biology and Health), Springer.

Zuur, A.F., Ieno, E.N., & Elphick, C.S. (2010). A protocol for data exploration to avoid common statistical problems. Methods in Ecology and Evolution, 1(1), 3-14.
